# 

**Published:** 1857-04

**Authors:** 


### RECEIPTS ON SUBSCRIPTIONS,

DURING MARCH, 1857.

**ILLINOIS.**

| | | |
|---|---|---|
| Dr. E. Smith, | Vol. 6, | $2.00 |
| " A. Hard, | Vol. 5 & 6, | 4.00 |
| " H. C. Daniels, | Vol. 6, | 2.00 |
| " J. F. Marrh, | " 6, | 2.00 |
| " J. P. Anthony, | Vol. 5 & 6, | 4.00 |
| " S. T. Brooks, | Vol. 2, 3, 4, 5, | 8.00 |
| " S. Thompson, | Vol. 4 & 5, | 4.00 |
| " G. V. Ewing, | Vol. 6, | 2.00 |
| " H. Wardner, | " 6, | 2.00 |
| " A. Berehleman, | " 6, | 2.00 |
| " E. Y. Nichols, | " 6, | 2.00 |
| " A. L. McArthur, | " 5, | 2.00 |
| " Simonton, | Vol. 4 & 5, | 4.00 |
| " F. K. Bailey, | Vol. 5, | 2.00 |
| " W. H. J. Davis, | " 5, | 2.00 |
| " J. F. Daggett, | " 5, | 2.00 |
| " Wm. Hawley, | " 5, | 2.00 |
| " A. F. Hand, | " 5, | 2.00 |
| " P. Kirwan, | " 6, | 2.00 |
| " C. Hard, | " 5, | 2.00 |
| " T. Hay, | Vol. 4 & 5, | 4.00 |
| " R. McArthur, | Vol. 5, | 2.00 |

**ILLINOIS—Continued.**

| | | |
|---|---|---|
| " F. Bry, | Vol. 5, | 2.00 |
| " Lescher & Miller, | Vol. 5 & 6, | 5.00 |
| " H. C. Morey, | Vol. 6, | 2.00 |
| " L. D. Glazelrook, | " 6, | 2.00 |
| " J. Newton, | " 6, | 2.00 |
| " H. Hall, | " 6, | 2.00 |
| " Geo. Higgins, | " 6, | 2.00 |
| " J. D. Arnold, | Vol. 4 & 5, | 4.00 |
| " E. Andrew, | " 4 & 5, | 4.00 |
| " Ed. Dickinson, | " 4 & 5, | 4.00 |
| " J. McCoy, | " 5 & 6, | 4.00 |
| " J. Petrie, | " 4 & 5, | 4.00 |

**INDIANA.**

| | | |
|---|---|---|
| Dr. M. L. Martin, | Vol. 6, | $2.00 |
| " W. M. Goodwin, | " 6, | 2.00 |

**MICHIGAN.**

| | | |
|---|---|---|
| Dr. Alex. DeArmand, | Vol. 6, | $2.00 |
| " White & Powers, | " 6, | 1.00 |

**WISCONSIN.**

| | | |
|---|---|---|
| Dr. C. Marsh, | Vol. 5, | $2.00 |

